# Noninvasive Mechanical Ventilation in Patients with Viral Pneumonia-Associated Acute Respiratory Distress Syndrome: An Observational Retrospective Study

**DOI:** 10.1155/2023/1819087

**Published:** 2023-02-01

**Authors:** Lu-lu Chen, Heng Weng, Hong-yan Li, Xin-hang Wang

**Affiliations:** ^1^Department of Respiratory Diseases, People' Hospital Affiliated to Fujian University of Traditional Chinese Medicine, Fuzhou 350009, China; ^2^Department of Critical Care Medicine, People' Hospital Affiliated to Fujian University of Traditional Chinese Medicine, Fuzhou 350009, China; ^3^Department of Respiratory Diseases, Fuzhou Pulmonary Hospital of Fujian, Fuzhou 350008, China

## Abstract

**Objectives:**

Appropriate mechanical ventilation may change the prognosis of patients with viral pneumonia-associated acute respiratory distress syndrome (ARDS). This study aimed to identify the factors associated with the success of noninvasive ventilation in the management of patients with ARDS secondary to respiratory viral infection.

**Methods:**

In this retrospective cohort study, all patients with viral pneumonia-associated ARDS were divided into the noninvasive mechanical ventilation (NIV) success group and the NIV failure group. The demographic and clinical data of all patients were collected. The factors associated with the success of noninvasive ventilation were identified by the logistic regression analysis.

**Results:**

Among this cohort, 24 patients with an average age of 57.9 ± 17.0 years received successful NIVs, and NIV failure occurred in 21 patients with an average age of 54.1 ± 14.0 years. The independent influencing factors for the success of the NIV were the acute physiology and chronic health evaluation (APACHE) II score (odds ratio (OR): 1.83, 95% confidence interval (CI): 1.10–3.03) and lactate dehydrogenase (LDH) (OR: 1.011, 95% CI: 1.00–1.02). When the oxygenation index (OI) is <95 mmHg, APACHE II > 19, and LDH > 498 U/L, the sensitivity and specificity of predicting a failed NIV were (66.6% (95% CI: 43.0%–85.4%) and 87.5% (95% CI: 67.6%–97.3%)); (85.7% (95% CI: 63.7%–97.0%) and 79.1% (95% CI: 57.8%–92.9%)); (90.4% (95% CI: 69.6%–98.8%) and 62.5% (95% CI: 40.6%–81.2%)), respectively. The areas under the receiver operating characteristic curve (AUC) of the OI, APACHE II scores, and LDH were 0.85, which was lower than the AUC of the OI combined with LDH and the APACHE II score (OLA) of 0.97 (*P*=0.0247).

**Conclusions:**

Overall, patients with viral pneumonia-associated ARDS receiving successful NIV have lower mortality rates than those for whom NIV failed. In patients with influenza A-associated ARDS, the OI may not be the only indicator of whether NIV can be used; a new indicator of NIV success may be the OLA.

## 1. Introduction

From the Spanish influenza of 1918 to the influenza outbreak in Hong Kong in 1968, the 20th century had three influenza A pandemics that brought unprecedented disasters to human populations, with the total number of deaths from influenza exceeding those of World War I. During the spring of 2013, a novel and highly virulent avian-origin influenza A subtype virus, H7N9, emerged among humans in eastern China. Overall, 1,161 people were infected by the end of January 2017, resulting in 433 deaths, a death rate of 37.3%. Infection with influenza A virus (H1N1) or avian influenza virus (H7N9) often leads to viral pneumonia that develops rapidly into acute respiratory distress syndrome (ARDS) [1–3]. As such, ARDS is associated with high mortality rates and is the leading cause of death from viral pneumonia.

Mechanical ventilation provides adequate respiratory support for patients with ARDS, but an inappropriate mechanical ventilation strategy can increase mortality [4, 5]. Several studies have shown that ARDS has a higher mortality rate when endotracheal intubation is needed [6, 7], and simultaneous complications associated with mechanical ventilation may also cause death in patients with ARDS [8].

Noninvasive ventilation (NIV) has been recommended as the first choice of ventilatory support for the management of patients with acute exacerbations of chronic obstructive pulmonary disease [9] and cardiogenic pulmonary edema [10]. Application of NIV in patients with ARDS may help to reduce both the intubation rate and its potential complications, which may ultimately affect the outcomes [11]. However, NIV has been used in a broad selection of ARDS patients [12, 13], and many problems remain. The subgroups of patients with ARDS who are most likely to benefit from NIV remain unclear [14, 15]. Although some factors leading to failed NIV in patients with ARDS are better understood after its consistent use, relatively few patients with influenza-associated ARDS have been studied to date [16, 17]. Also, clinical data are needed to support the timing of NIV therapy for patients with viral pneumonia-associated ARDS. The purpose of this study was to identify the factors associated with the success of NIV in the management of patients with ARDS secondary to respiratory viral infection.

## 2. Materials and Methods

### 2.1. Study Population

In this retrospective monocenter observational study, we reviewed the clinical data of patients who were admitted to the 16-bed intensive care unit (ICU) of the Fuzhou Pulmonary Hospital of Fujian, China, between October 1, 2013, and March 31, 2019. This hospital is the only one designated for treating respiratory infectious diseases in Fujian Province.

Patients who met the following inclusion criteria were eligible to be included in this study: (1) ≥18 years of age; (2) tested positive for influenza A. After admission, respiratory specimens (nasopharyngeal swabs, sputum, or endotracheal aspirates) were collected daily to determine the amount of influenza viral RNA by polymerase chain reaction (PCR) analysis. Infection with influenza A was confirmed by testing a sample acquired via nasopharyngeal swab or bronchoalveolar lavage and obtaining a positive result in a probe-based reverse transcriptase-PCR for H1N1, as previously described [18]; (3) diagnosed by the 2011 ARDS Berlin definition [19]. The exclusion criteria were as follows: (1) patients who died before passing from NIV to mechanical ventilation; (2) patients who had been intubated on admission; (3) patients who can be treated with high-flow nasal cannula oxygen therapy.

### 2.2. Data Acquisition

All patient-related medical data regarding demographics, comorbidity, clinical examinations, radiological findings, microbiologic investigations, and therapeutic management collected later than 3 h after the ICU admission were collected. All laboratory tests were extracted within 1 h of the ICU admission. Laboratory indexes were evaluated in duplicate samples in the hospital's clinical laboratory. During the study period, all consecutive patients underwent a microbial etiology screening in the respiratory tract within 72 h following their ICU admission. Mortality was defined as death from any cause within 30 d of hospitalization. Pneumonia severity was assessed by the Murray lung injury score. The acute physiology and chronic health evaluation (APACHE) II score was used to assess the severity of the disease, and the sequential organ failure assessment (SOFA) score was used to assess organ dysfunction. Additionally, the APACHE II score, Murray lung injury score, and SOFA score were recorded within 24 h after patients were admitted to the ICU. The medical records were independently reviewed by two physicians.

The standard for respiratory tract specimen collection was as follows: (1) all patients were required to provide sputum samples before using antibiotics or antiviral drugs. If the patient needed invasive mechanical ventilation within 24 h of admission, an alveolar lavage fluid sample was taken; (2) patients were required to rinse their mouths with clear water three times before sputum retention. Sputum induction by 3%–5% sodium chloride atomization was used in patients with expectoration difficulties; (3) all sputum and alveolar lavage fluid samples were stored in sterile containers.

### 2.3. Definitions

Nosocomial infection was defined as follows: (a) pulmonary bacterial or fungal infection was confirmed by histology; (b) pneumonia manifestations can be seen in imaging, and microorganisms isolated by blood culture or chest fluid culture were consistent with sputum culture results; (c) after two weeks of treatment, the body temperature was normal, and the respiratory symptoms, such as cough and sputum, had improved. However, the appearance or worsening of dyspnea and cough, which may be associated with fever or chest pain, was accompanied by changes in radiological images; (d) new-onset purulent sputum, change in sputum character, or increased respiratory secretions or suctioning requirement.

Successful NIV was defined as continued noninvasive ventilator-assisted breathing without intubation after 2 h of noninvasive ventilator-assisted therapy. Failed NIV was defined as qualifying for intubation after 2 h of noninvasive ventilator-assisted therapy.

### 2.4. Treatments

All enrolled patients received NIV therapy, which included both continuous positive airway pressure (CPAP) and intermittent positive pressure breathing. No sedative drugs were administered during NIV treatment. All NIV was performed by a Philips V60. (CPAP, i.e., expiratory positive airway pressure, was set at 10 cmH2O, and FiO2 was set according to the patient's need). After 2 h, all patients were assessed for their oxygen index (OI), level of respiratory muscle activity, mind, and hemodynamic readings. The standards for endotracheal intubation were as follows [20, 21]: (1) failure to maintain an arterial oxygen pressure (PaO_2_) ≥ 60 mmHg, with persistent dyspnea, tachypnea, or activation of accessory respiratory muscles (auxiliary respiratory muscles score ≥ 3); (2) development of conditions necessitating endotracheal intubation to protect the airways (coma or seizure disorders) or to manage copious tracheal secretions; (3) any hemodynamic or electrocardiographic instability (i.e., systemic hypotension lasting ≥1 h despite fluid resuscitation); (4) inability to correct dyspnea or inability to tolerate the mask.

### 2.5. Statistical Analysis

All statistical analyses were performed using SPSS™ Statistics version 15.0 (SPSS™, Chicago, IL, USA) and MedCalc version 15.2. All continuous variables were evaluated for normal distribution using the Kolmogorov–Smirnov test. Categorical variables are presented as a percentage of the total. For between-group comparisons, the Student's *t*-test and the Wilcoxon test were used for continuous variables, and the chi-squared or Fischer's exact test was used for categorical data. The correlation of variables was obtained using Spearman's or Pearson's rank correlation coefficient. Parametric data are presented as the mean ± standard deviation, and nonparametric data are presented as the median and interquartile range (IQR). Significance was defined as *P* < 0.05. Based on the logistics model, combining predictors, or probabilities, were applied to establish the empirical and binormal model of the receiver operating characteristic (ROC) curve.

## 3. Results

### 3.1. Demographic and Clinical Data

**Figure 1:**
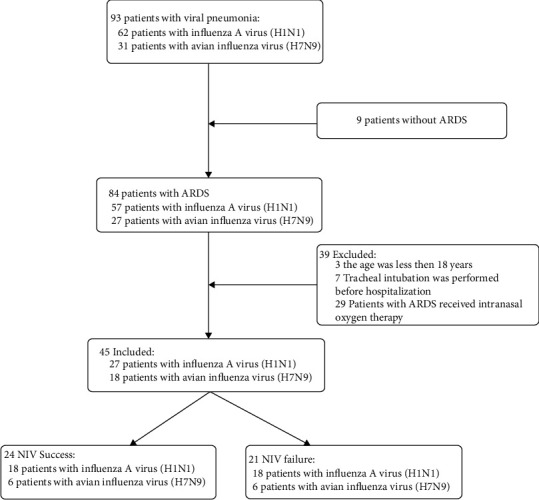
Flowchart of the study.

A total of 294 patients were diagnosed with H1N1 or H7N9 and treated in our hospital between October 1, 2013, and March 31, 2019. Among them, 93 patients were diagnosed with H1N1 or H7N9-related pneumonia. In 84 patients, ARDS developed, and 29 of them received intranasal oxygen therapy. In three cases of ARDS, the patient was <18 years old. Tracheal intubation was performed in seven patients before hospitalization. Finally, 45 patients were included in this study, and their data were used for analysis. The recruitment flowchart is shown in Figure 1.

The average age of this cohort was 56.2 ± 15.6 years, and 80.0% of the patients were men. The mean body mass index (BMI) was 25.5 ± 2.9 kg/m^2^, and only two (4.4%) patients were obese (BMI >30 kg/m^2^). On admission, the mean APACHE II score, SOFA score, and Murray's lung injury score were 22.8 ± 9.3, 6.1 ± 3.5, and 3.2 ± 0.6, respectively. The mean time between onset of the symptom and presentation was 5.7 ± 2.1 d. The medium ICU stay was 14 d (IQR 10–24 d).

Among the 45 patients, 24 patients received successful NIV (successful NIV group), comprising 6 patients with H7N9 and 18 patients with influenza A (H1N1), with an average age of 57.9 ± 17.0 years. Patients who received NIV initially but needed subsequent intubation were included in the failed NIV group. There were 21 patients in the failed NIV group, comprising 12 patients with H7N9 and 9 patients with influenza A (H1N1), with an average age of 54.1 ± 14.0 years (Figure 1). The baseline characteristics of the two groups are displayed in Table 1.

### 3.2. Laboratory Indexes, Clinical Symptoms, and Physical Examination

The duration from symptom onset to the initiation of antiviral agents was not significantly different between successful and failed NIV groups (6.0 ± 2.3 d and 5.3 ± 1.8 d, respectively, *P*=0.336). The duration of the fever in the hospital was significantly different between the successful and failed NIV groups (2 d (IQR 0–5 d) and 5 d (IQR 2–9 d), respectively, *P*=0.007).

The mean OI for successful NIV and failed NIV was 164.0 ± 71.4 mmHg and 85.9 ± 33.1 mmHg, respectively (*P* < 0.001). In the successful NIV group, the distributions of OI were as follows: 13 patients (13/24; 54.2%) had OI > 150 mmHg, 6 patients (6/24; 25.0%) had OI of 100–150 mmHg, and 5 patients (5/24; 20.8%) had OI < 100 mmHg. In the unsuccessful NIV group, no patients had OI > 150 mmHg, 7 patients (7/21; 33.3%) had OI of 100–150 mmHg, and 14 patients (66.7%) had OI < 100 mmHg.

The median serum levels of lactate dehydrogenase (LDH) and blood urea nitrogen of the failed NIV group were markedly increased compared with the successful NIV group (826.0 (IQR 629.5–1446.0) vs. 447.0 (IQR 383.2–680.5) U/L, respectively, *P* < 0.001; and 5.25 (IQR 3.9–6.8) vs. 7.08 (IQR 5.1–14.2) mmol/L, respectively, *P*=0.032). No significant differences were found in other laboratory indexes between the two groups, including white blood cell count, C-reactive protein, and procalcitonin (Table 2).

### 3.3. Nosocomial Infection Rate and Prognosis

After 5 d of admission, a total of 10 patients had a nosocomial infection (3 in the NIV success group, 7 in the NIV failure group). Lower respiratory tract specimens for bacterial culture were obtained in none of the patients, while lower respiratory tract specimens for fungi culture were obtained in three patients (12.5%; all with *Aspergillus*) in the successful NIV group. In the failed NIV group, lower respiratory tract specimens for bacterial culture were obtained in five patients (5/21, 23.8%), comprising one case each of *Sphingomonas oligosaccharides*, *Xanthomonas indolens* combined with *Pseudomonas aeruginosa*, *Stenotrophomonas maltophilia*, *Staphylococcus aureus*, and *Acinetobacter baumannii*. Lower respiratory tract specimens for fungi culture were obtained in two patients (2/21, 9.5%; *Aspergillus* combined with trichosporosis in one case, and *Aspergillus* in another case). Four out of ten patients with nosocomial infections died, 1 (1/24; 4.2%) in the NIV success group and 3 (3/21; 14.3%) in the NIV failure group (*P*=0.234).

**Figure 2:**
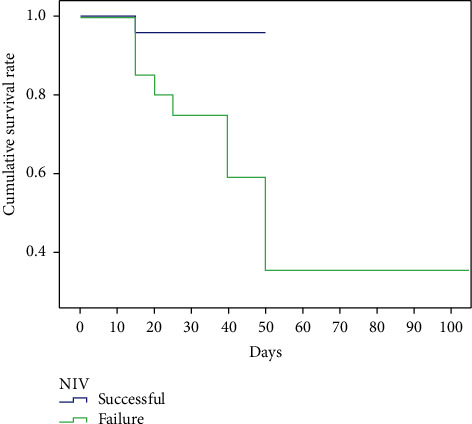
Survival rate of the two groups.

Among the 24 patients with successful NIV, one died because of hemodynamic abnormalities after 3 d of NIV treatment. Rejection of intubation by dependents was the cause of death. Reasons for death among the failed NIV group included shock (*n* = 8; 38.1%) and pulmonary embolism (*n* = 1; 4.7%). Mortality data were obtained for the successful NIV group, and the mortality rate was lower than in the failed NIV group (4.1% vs. 42.8%, *𝒳*^2^=7.591; *P*=0.003) (Figure 2).

### 3.4. Logistic Regression Analysis

The parameters that were significantly different (*P* < 0.05) between the two groups were included in the logistic regression analysis, including the APACHE II score, the SOFA score, Murray's lung injury score (Table 1), the duration of fever in hospital, the heart rate at admission, the OI levels, the BUN levels, the LDH levels, and the median length of ICU stay (Table 2). Logistic regression analysis revealed that LDH (odds ratio (OR): 1.01, 95% CI: 1.00–1.02; *P*=0.034) and the APACHE II score (OR: 1.83, 95% CI: 1.10–3.03; *P*=0.019) were the independent influencing factors for a successful NIV.

### 3.5. Predictive Factors of Noninvasive Mechanical Ventilation

**Figure 3:**
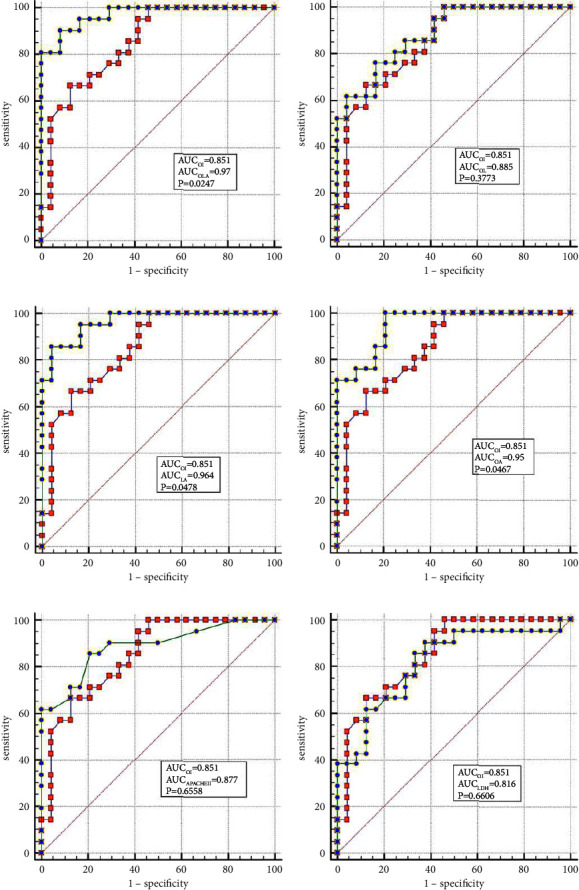
(a) Combining the area under the ROC curve of APACHE II with OLA (AUC = 0.887 vs. AUC = 0.970; *P*=0.084). (b) Combining the area under the ROC curve of LDH with OLA (AUC = 0.816 vs. AUC = 0.970; *P*=0.013). (c) Combining the area under the ROC curve of OI with OLA (AUC = 0.851 vs. AUC = 0.970; *P*=0.024). (d) Combining the area under the ROC curve of OL with OLA (AUC = 0.885 vs. AUC = 0.970; *P*=0.042). (e) Combining the area under the ROC curve of OA with OLA (AUC = 0.950 vs. AUC = 0.970; *P*=0.310) (f) Combining the area under the ROC curve of LA with OLA (AUC = 0.964 vs. AUC = 0.970; *P*=0.479).

The best factors associated with successful NIV were identified with ROC curves. The areas under the ROC curve (AUC) of the OI, APACHE II scores, and LDH were 0.851 (95% CI: 0.74–0.96), 0.887 (95% CI: 0.78–0.98), and 0.816 (95% CI: 0.69–0.94), respectively. The AUC of OI combined with LDH and APACHE II (OLA) was 0.970, which was higher than the OI AUC of 0.851 (*P*=0.024). When the OI is <95.0 mmHg, the APACHE II score is >19.0, and LDH is  > 498.0 U/L, the sensitivity and specificity of predicting a failed NIV were (66.6%, (95% CI: 43.0%–85.4%) and 87.5% (95% CI: 67.6%–97.3%)); (85.7% (95% CI: 63.7%–97.0%) and 79.1% (95% CI: 57.8%–92.9%)); (90.4% (95% CI: 69.6%–98.8%) and 62.5% (95% CI: 40.6%–81.2%)), respectively, (Figures 3(a)–3(f)).

## 4. Discussion

Patients infected with H1N1 and H7N9 are found every year in Fujian Province [22]. The distribution of cases by month of onset was as follows: cases began to appear in November, increased in a stepwise manner, reached a peak incidence in January of the following year, and decreased until there were no cases after May [23]. In the present study, we analyzed the clinical data of patients with viral pneumonia-associated ARDS and attempted to determine the influencing factors associated with the success and failure of NIV treatment. Twenty-four patients received successful NIVs, comprising 6 with H7N9 and 18 with H1N1. Twenty-one patients experienced NIV failure. The mortality data in the present study showed that the group with successful NIV had a lower mortality rate than the failed NIV group. The independent influencing factors for a successful NIV were shown to be LDH and the APACHE II scores.

In the present study, OI appeared to be associated with successful NIV use, but the results were not significant. According to the Berlin definition, in 2011, patients with ARDS were divided into mild, moderate, and severe cases, according to the OI [19]. Currently, the OI is still one of the most important indexes for evaluating the effectiveness of mechanical ventilation for the treatment of ARDS. However, there is no unified standard for the use of mechanical ventilation in Asia or western countries. Most clinical studies and reports set the OI at 200 mmHg or 150 mmHg as a demarcation point for the possibility of NIV [24, 25]. Among the 24 patients with successful NIV, an OI <150 mmHg was obtained in 11 (11/24, 45.8%) patients. In clinical work, it was found that in patients with an auxiliary respiratory muscles score <3, even if the OI was <150 mmHg, most patients can be successfully treated with NIV [26]. In the present study, a risk prediction was made for the success of NIV, and an OI of 95 mmHg was found to be the critical point. It is believed that the combined evaluation of the auxiliary respiratory muscles score and the OI is more critical in determining whether tracheal catheterization should be performed. It is suggested that when the auxiliary respiratory muscles score is <3, invasive mechanical ventilation can be avoided in some patients with 200 > OI > 95 mmHg.

When lung tissue is destroyed, large amounts of LDH are released into the blood, resulting in significant increases in the concentration of serum LDH [27]. Mura et al. [28] reported that the level of serum LDH in mice with acute lung injury was significantly higher than in the control group and that the change in plasma LDH levels can occur earlier than the decreased oxygenation capacity. Patients with severe influenza A often die of ARDS or multiple organ failure, and significantly elevated LDH levels are a marker for multiple organ injury and may indicate a poor prognosis [29]. Higher APACHE II scores indicate that the condition is more severe, and the prognosis is poorer [30]. Logistic regression analysis confirmed that LDH and the APACHE II scores are the independent influencing factors for the success of the NIV. In the present study, the AUCs of the OI, LDH, and APACHE II scores were analyzed individually and jointly, showing that the AUC of OLA was the highest among all indicators and that the values were closer to unity, indicating the highest predictive value.

In the present study, after 5 d of admission, no lower respiratory tract specimens for bacterial culture were obtained in patients with successful NIV, but five (5/21; 23.8%) patients in the failed NIV group had coinfection with *Aspergillus*. According to previous reports, on admission, patients with influenza A had bacterial coinfection (25%; mostly *Streptococcus pneumonia*) [31]. A research report from China indicated that the incidence of nosocomial pneumonia from severe influenza is as high as 33% [32]. Documents in South Korea reported that once patients with severe influenza have a nosocomial infection, the mortality rate may be as high as 71.4% [33].

The influenza virus can damage airway epithelial cells, which leads to structural and functional abnormalities in the airways. Advantages of NIV in the treatment of patients with ARDS include avoiding structural damage to the respiratory tract caused by invasive mechanical ventilation and complications, such as ventilator-associated pneumonia [34, 35]. van de Veerdonk et al. [36] reported a series of nine cases of invasive aspergillosis among 40 critically ill patients with influenza A (H1N1) infection (9/40; 23%) with very high mortality (61%). Wauters et al. [37] reported that invasive pulmonary aspergillosis was diagnosed in 23% of critically ill patients with influenza A (H1N1) virus infection within a median of 3 d after the ICU admission and that the use of corticosteroids 7 d before the ICU admission is an independent risk factor for fungal superinfection. In the present study, *Aspergillus* was detected in five patients after 5 d of admission, which suggests an association with the use of corticosteroids before admission.

During NIV, the expired tidal volume (Vte) is the result of both the pressure support level delivered by the ventilator and the respiratory muscle pressure generated by the patient. In a recent clinical trial, Needham et al. [38] emphasized the importance of controlling Vte at the very onset of ARDS, demonstrating that higher tidal volumes after ARDS onset were associated with a greater risk of ICU mortality. The impact of Vte on NIV outcomes is driven mainly by hypoxemia, mostly occurring in patients with the worst lung injury. Failure of NIV was also predicted accurately by a mean Vte >9.5 mL/kg predicted body weight recorded over the first four cumulative h of NIV [17]. In the present study, no significant differences were found in tidal volumes between the two groups.

### 4.1. Limitations

This study has several limitations. First, the study analyzed data retrospectively, which limits the inference of cause. Second, it is a single-center cohort study with a relatively small sample size, and the data may not be very representative of the population studied; results may also be limited to this population only and may not be generalized to other populations. Also, the data did not include how many hours may have passed between changing from NIV to invasive mechanical ventilation, which may bias the results. Further prospective multicenter studies are needed to confirm the results of the present study.

## 5. Conclusion

To sum up, patients with viral pneumonia-associated ARDS receiving successful NIV have lower mortality rates than those receiving failed NIV. In patients with influenza A-associated ARDS, OI may not be the only indicator to determine whether NIV can be used. OLA may be a new indicator of NIV success.

## Figures and Tables

**Table 1:** Demographic and clinical characteristics of 45 patients with influenza A-associated acute respiratory distress syndrome.

	NIV success (*n* = 24)	NIV failure (*n* = 21)	*P* value
*Patient characteristics*			
Age (years)	57.9 ± 17.0	54.1 ± 14.0	0.427
Male gender	20 (83.3%)	16 (76.1%)	0.713
BMI (kg/m^−2^)	25.5 ± 2.5	25.6 ± 3.5	0.845
*Underlying diseases*			
Diabetes	1 (4.1%)	5 (23.8%)	0.083^a^
Hypertension	8 (33.3%)	9 (42.8%)	0.552
Chronic obstructive pulmonary disease (COPD)	1 (4.1%)	0 (0.0%)	1.000^a^
Coronary heart disease	3 (12.5%)	1 (4.7%)	0.611^a^
Lower limbs varicosity	2 (8.3%)	0 (0.0%)	0.491^a^
Schizophrenia	1 (4.1%)	1 (4.7%)	1.000^a^
Renal calculus	2 (8.3%)	0 (0.0%)	0.491^a^
Hepatocirrhosis	1 (4.1%)	0 (0.0%)	1.000^a^
Parkinson's disease	1 (4.1%)	0 (0.0%)	1.000^a^
Tricuspid valve replacement (TVR)	1 (4.1%)	0 (0.0%)	1.000^a^
*Types of influenza viral*			
H7N9	6 (25.0%)	12 (57.1%)	0.037
H1N1	18 (75.0%)	9 (42.8%)	—
*Disease score*			
APACHEII	16.8 ± 3.9	29.6 ± 9.2	<0.001
SOFA	4.0 ± 1.3	8.5 ± 3.7	<0.001
Murray	2.8 ± 0.5	3.7 ± 0.3	<0.001

Note. APACHE II = acute physiology and chronic health evaluation II; SOFA = sequential organ failure assessment. ^a^Calculated using Fischer's exact test.

**Table 2:** Laboratory indexes, clinical symptoms, and physical examination of 45 patients with influenza A-associated acute respiratory distress syndrome.

	NIV success (*n* = 24)	NIV failure (*n* = 21)	*P* value
*Symptom*			
Cough	24 (100%)	21 (100%)	—
Expectoration	24 (100%)	21 (100%)	—
Hemoptysis	7 (29.1%)	11 (52.3%)	0.138^a^
Fever	24 (100%)	20 (95.2%)	0.467^a^
Dyspnea	21 (87.5%)	21 (100%)	0.236^a^
Body aches	8 (33.3%)	4 (19.0%)	0.329^a^
Headache	2 (8.3%)	4 (19.0%)	0.396^a^
Melena	1 (4.1%)	1 (4.1%)	1.000^a^
Vomit	1 (4.1%)	0 (0.0%)	1.000^a^
Anorexia	1 (4.1%)	2 (9.5%)	0.592^a^
*Clinical features*			
Days with fever before admission	5.6 ± 2.3	5.3 ± 1.8	0.655^b^
Days with dyspnea before admission	4.5 ± 2.4	4.1 ± 1.7	0.629^b^
The mean time from symptom onset to presentation (days)	6.0 ± 2.3	5.3 ± 1.8	0.336^b^
Aximum body temperature (°C)	39.1 ± 0.8	39.0 ± 0.8	0.514^b^
The duration of fever in hospital (days)	2 (0–5)	5 (2–9)	0.007^c^
*Vital signs*			
Heart rate in admission (cycles/min)	99 ± 12	111 ± 14	0.006^b^
Systolic pressure (mmHg)	125 ± 18	130 ± 20	0.445^b^
Diastolic pressure (mmHg)	76 ± 12	78 ± 17	0.633^b^
OI (mmHg)	164.0 ± 71.4	85.9 ± 33.1	<0.001^b^
*Laboratory index*			
W.B.C count (10^9^/L)	5.0 ± 2.1	6.1 ± 4.6	0.335^b^
Lymphocyte count (10^9^/L)	0.6 ± 0.4	0.5 ± 0.2	0.276^b^
Platelet count (10^9^/L)	148.0 ± 46.9	154.6 ± 62.0	0.686^b^
C-reactive protein (mg/L)	131.9 ± 68.7	162.6 ± 74	0.157^b^
Procalcitonin (ng/mL)	1.5 ± 2.4	3.5 ± 5.8	0.134^b^
APTT	34.9 ± 5.3	36.9 ± 10.1	0.399^b^
Fibrinogen (g/L)	3.4 ± 0.9	3.4 ± 1.0	0.987^b^
ALT (U/L)	40.5 (33.2–86.0)	60.0 (38.0–96.1)	0.311^c^
AST (U/L)	57.5 (48.9–98.0)	103.0 (59.0–194.0)	0.090^c^
ALP (U/L)	77.5 (61.2–106.5)	93.0 (80.5–137.8)	0.072^c^
BUN (mmol/L)	5.2 (3.9–6.8)	7.0 (5.1–14.2)	0.032^c^
Cr (*μ*mol/L)	85.2 (68.1–96.9)	87.4 (68.0–146.1)	0.363^c^
LDH (U/L)	447.0 (383.2–680.5)	826.0 (629.5–1446.0)	<0.001^c^
CK (U/L)	149.0 (108.5–813.5)	187.0 (58.5–1138.0)	0.593^c^
CK-MB (U/L)	13.8 (8.0–20.4)	15.0 (10.5–35.3)	0.187^c^
*Ventilator parameters*			
Vte (ml/kg)	7.2 ± 1.5	7.8 ± 2.3	0.380^b^
The median length of ICU stay (days)	10.0 (6.2–14.0)	24.5 (13.5–40.0)	<0.001^c^

Note. OI = oxygenation index; APTT =  activated partial thromboplastin time; ALT =  alanine aminotransferase; AST =  aspartate aminotransferase; ALP = alkaline phosphatase; BUN = blood urea nitrogen; Cr = creatinine; LDH = lactate dehydrogenase; CK = creatine kinase; CK-MB = creatine kinase isoenzyme; Vte = expired tidal volume (calculated every 2 h and the average value was used for analysis). ^a^Calculated using the chi-square test; ^b^calculated using student's *t* test; ^c^calculated using the Wilcoxon rank sum test.

## Data Availability

The datasets used and/or analyzed during the current study are available from the corresponding author on reasonable request.
